# The Role of INSR & Diabetes in Polycystic Ovarian Syndrome

**Published:** 2018-10

**Authors:** Afrouz KHAZAMIPOUR, Azim NEJATIZADEH, Eghbal EFTEKHAARI TASNIM

**Affiliations:** Molecular Medicine Research Center, Hormozgan Health Institute, Hormozgan University of Medical Sciences, Bandar Abbas, Iran

## Dear Editor-in-Chief

Polycystic ovarian syndrome (PCOs) was first reported as Stein and Leventhal syndrome in 1935 (1). PCO as a multifactorial disease with both multiple genetic components and environmental factors with a variety of clinical manifestation and genetic heterogeneity is a common endocrine disorder among women of reproductive age affecting 5%–10% of the population (2). Genetic basis of PCO as a complex trait is not well known (3). PCO is heterogeneous endocrine disorder characterized by chronic anovulation, excess androgen production, overweightness and obesity, insulin resistance and increased susceptibility to diabetes mellitus type II (4).

**Fig. 1:**
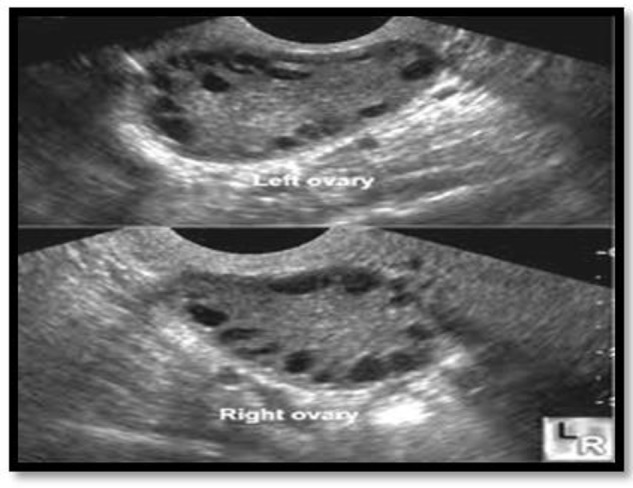
This sonography displaying polycystic ovaries

In this cross-sectional case-control study, 180 women with 18–40 yr old age and diagnosis of PCO were confirmed by two positive signs out of three Rotterdam diagnostic criteria (irregular menarche, hyperandrogenism clinical signs for example acne, hirsutism, alopecia, and ovarian sonography displaying two cystic ovaries carrying more than 12 cysts with 2–9 mm diameter) (5) (Fig. 1).

Even though a great number of genomic variants have been observed to be associated with PCOS, no single candidate gene has appeared as an indisputable biomarker so far.

Insulin receptor gene (INSR) has 22 exons with 120 kb of length located on chromosome 19. Insulin and insulin receptors in ovaries have several important impacts on ovaries; androgen secretion stimulation, steroidogenic response creation of ovaries to LH, FSH hormones and process harness of apoptosis in ovarian follicles which leads to cyst formation. The action of Insulin on ovarian also might cause hyper-androgenic inducing, lack of ovulation and cyst creation in PCOS patients (6). Insulin resistance disease is the main feature of PCOS pathophysiology (7).

Exon 17 insulin receptor gene showed significant association with PCOS (especially with insulin reduced sensitivity) (8). Chinese population were studied, frequency of the exon 17 insulin receptor gene in PCOS patient was reported more than control group (51.4% > 38.3%), in PCOS women was reported almost 4 times more than healthy women (8). However, in our study, no significant differences between the two groups of case and control were observed (*P*<0.05).
